# Quality of Life Assessment in Non-melanoma Skin Cancers and Comparison of the Quality of Life Between Psoriasis and Vitiligo Patients

**DOI:** 10.7759/cureus.90783

**Published:** 2025-08-22

**Authors:** Ferdi Öztürk, Serap Öztürkcan, Erhan Eser, Mustafa Turhan Sahin

**Affiliations:** 1 Department of Dermatology, Faculty of Medicine, Bursa Uludag University, Bursa, TUR; 2 Department of Dermatology, Faculty of Medicine, Manisa Celal Bayar University, Manisa, TUR; 3 Department of Public Health, Faculty of Medicine, Manisa Celal Bayar University, Manisa, TUR

**Keywords:** non-melanoma skin cancers, psoriasis, quality of life, skin cancers, vitiligo

## Abstract

Objective

Despite the increase in incidence of non-melanoma skin cancers (NMSC), deaths are relatively rare. The goal of treatment is to prevent the recurrence of the disease and to increase the quality of life (QoL). The QoL and the cost of treatment are becoming more important treatment goals, as nowadays NMSC are rarely life-threatening. This study aims to assess and compare the QoL in patients with NMSC, psoriasis, and vitiligo using three validated QoL instruments.

Methods

A total of 150 patients were included in this study, with 50 patients each diagnosed with NMSC, psoriasis, and vitiligo. The QoL was assessed using the European Organization for Research and Treatment of Cancer Core QoL Questionnaire (EORTC QLQ-C30), the Dermatology Life Quality Index (DLQI), and the World Health Organization Quality of Life Scale Short Form (WHOQOL-BREF). Sociodemographic data and disease severity were also recorded.

Results

The study found that NMSC and psoriasis patients had similar QoL scores, with both groups showing more significant impairment in QoL than vitiligo patients. Psoriasis patients experienced greater deterioration in daily activities and personal relationships, while QoL in work, school, and treatment domains was worse in both NMSC and psoriasis patients compared to vitiligo patients. A negative correlation was observed between disease duration and the physical health domain subscale of the WHOQOL-BREF, as well as the work and school subscale of the DLQI, in vitiligo patients; and between disease duration and the work and school subscale of the DLQI in NMSC patients.

Conclusion

The findings underscore the significant impact of NMSC on patients’ QoL, highlighting the importance of comprehensive management strategies in these conditions. The similarity in QoL scores obtained across all groups using DLQI and WHOQOL-BREF suggests that the WHOQOL-BREF, a general health QoL scale, can be effectively used alongside the DLQI, a dermatology-specific QoL scale, to assess QoL in dermatologic diseases.

## Introduction

Non-melanoma skin cancer (NMSC) is currently the most common malignancy. The term is used to describe a variety of tumors, but it mainly refers to basal cell carcinoma (BCC) and squamous cell carcinoma (SCC), which account for a large proportion of these neoplasms [1].

BCC and SCC have a low mortality rate, but because the lesions usually affect the skin of the head and neck, there may be significant morbidity from disfigurement [2]. The presence and subsequent treatment of NMSC can cause disfigurement, sometimes leading to physical and psychosocial dysfunction. Consequently, quality of life (QoL) is an important endpoint to consider in the treatment of NMSC.

The European Organization for Research and Treatment of Cancer Core QoL Questionnaire (EORTC QLQ-C30) is the most commonly used QoL tool in oncology. It was developed by Aaronson et al. and consists of 30 items and two subscales: functional and symptom scales. The functional subscale consists of six sub-dimensions: physical (questions 1-5), role (questions 6 and 7), cognitive (questions 20 and 25), emotional (questions 21-24), social (questions 26 and 27), and global quality of life (questions 29 and 30). The symptom subscale includes symptoms of fatigue (questions 10, 12, and 18), nausea and vomiting (questions 14 and 15), pain (questions 9 and 19), dyspnea (question 8), sleep disturbance (question 11), appetite loss (question 13), constipation (question 16), diarrhea (question 17), and financial impact (question 28). A high score on the scale indicates a high level of functional or global QoL and a high degree of symptom severity [3].

The EORTC QLQ-C30 was adapted into Turkish by Güzelant et al., and its validity and reliability were determined for the Turkish population [4]. We obtained permission from EORTC to use this tool in our study.

Dermatology Life Quality Index (DLQI) is a practical quality of life scale adapted into Turkish by Öztürkcan et al., and it can be used for all dermatological diseases. It consists of 10 questions designed to assess how the disease has affected the patient’s social and physical life over the past week. QoL deteriorates as the score increases [5,6]. This is a free-to-use tool and no permission is required for its use.

The World Health Organization Quality of Life Scale Short Form (WHOQOL-BREF) is a quality of life assessment tool developed by the World Health Organization to subjectively evaluate an individual’s quality of life in physical, social, and environmental domains. As the score increases, so does the quality of life [7]. We have obtained permission from the Principal Investigator of the relevant national center responsible for the Turkish version to use this tool in our study.

In this study, EORTC QLQ-C30, DLQI, and WHOQOL-BREF were administered to non-melanoma skin cancer cases, and DLQI and WHOQOL-BREF to psoriasis and vitiligo patients. There are many studies in the literature evaluating QoL in non-melanoma skin cancers (basal cell carcinoma and squamous cell carcinoma), but the combination of QoL scales we used to compare QoL is not available. In this study, we aimed to compare the QoL of non-melanoma skin cancers with the QoL of diseases such as psoriasis and vitiligo, which have been shown to severely affect QoL. We compared NMSC with psoriasis and vitiligo because, despite their different etiologies, all three diseases can cause visible skin lesions and carry a significant psychosocial burden. Assessing them in parallel allows the identification of both common and disease-specific impacts on quality of life, providing valuable insights for targeted patient management and support strategies.

This article was previously presented as an abstract at the annual scientific 20th Prof. Dr. A. Lütfü Tat Symposium in Turkey on November 16-20, 2011.

## Materials and methods

This prospective study was undertaken in the dermatology outpatient clinic of Manisa Celal Bayar University Hospital between October 1, 2010, to March 31, 2011. The study included 150 patients aged 18 and over, including 50 with a clinical and/or histopathological diagnosis of non-melanoma skin cancer, 50 with psoriasis, and 50 with vitiligo. In all groups, quality of life was assessed in the presence of active disease and prior to the initiation of disease-specific treatments. The study’s inclusion criteria were that patients be 18 years old or older and have no psychiatric diseases that could impair their QoL, whereas the exclusion criteria were that they be under 18 years old, who has an uncontrolled systemic disease that might affect quality of life and have psychiatric conditions. EORTC QLQ-C30, DLQI, and WHOQOL-BREF were administered to non-melanoma skin cancer cases, and DLQI and WHOQOL-BREF to psoriasis and vitiligo patients. For the assessment of DLQI and WHOQOL-BREF scores, a total of 150 participants were included, with 50 participants assigned to each of Group 1, Group 2, and Group 3. Sociodemographics, disease severity assessment (Psoriasis Area and Severity Index (PASI) for psoriasis), and comorbid health problem questionnaires were also administered to the study participants. Age, gender, marital status, occupation, education level, type of disease, localization of lesions, duration of disease, and comorbidities were recorded.

Prior to the study, approval was obtained from the Medical Ethics Committee of the Manisa Celal Bayar University Faculty of Medicine, confirming that the study was not objectionable in terms of medical ethics. Patients were informed about the study, and written informed consent was obtained.

The study data were evaluated with the SPSS Statistical Package Program for Windows 21.0 (IBM Corp., Armonk, NY). The normality of the data was assessed using the Shapiro-Wilk test. After basic descriptive statistics (percentage, frequency), groups were tested with analysis of variance (ANOVA) analysis of variance with post hoc Tukey B analysis and Mann-Whitney U test for continuous variables. For comparisons of groups (non-melanoma skin cancer, psoriasis, and vitiligo groups), Student’s t-test was used for continuous variables and chi-square test for categorical variables. In addition, Pearson correlation test was applied to examine the linear relationship between numerical variables. P<0.05 was considered statistically significant.

## Results

The study included 50 patients with non-melanoma skin cancer, 50 with psoriasis, and 50 with vitiligo. There was no difference between the patient groups in terms of gender (Chi-square=0.48; P=0.79). The mean ages of the patient groups (years) were 66.6±13.1 in the non-melanoma skin cancer group, 43.1±13.1 in the psoriasis group, and 43.6±14.0 in the vitiligo group. There was a statistically significant difference between the groups in terms of mean age (analysis of variance (ANOVA); P<0.001). The mean ages were similar in psoriasis and vitiligo patients, while the mean age in the non-melanoma skin cancer group was higher than the other two groups (post-hoc Tukey B). The mean duration of disease (months) in the patient groups was 22.2±27.6 in the non-melanoma skin cancer group, 129.5±122.9 in the psoriasis group, and 121±114.6 in the vitiligo group. There was a statistically significant difference between the groups in terms of mean disease duration (ANOVA; P<0.001). The mean duration of disease was similar in psoriasis and vitiligo patients, whereas the mean duration of disease was shorter in the non-melanoma skin cancer group than in the other two groups (post-hoc Tukey B).

Patients were regrouped according to their occupations as housewives, lower class (worker, farmer, secretary, driver), middle class (student, retired, civil servant, teacher, nurse, tradesman, journalist), and no statistical difference was found between the groups in terms of social class (Chi-square test; P=0.101). When the patients were grouped according to the education they received into middle school and below, high school, and above, a significant difference was found between the groups. In the non-melanoma skin cancer group, the number of people with secondary school and below education was higher than in psoriasis and vitiligo patients (Chi-square test; P<0.001). Lower education level may limit access to health information and awareness of preventive measures, potentially affecting timely diagnosis, treatment adherence, and ultimately quality of life.

When the comorbid systemic diseases in the patient groups (non-melanoma skin cancer, psoriasis, and vitiligo groups) were analyzed, 93 (62.0%) patients had no comorbid systemic disease, 41 (27.3%) patients had one systemic disease, 13 (8.7%) patients had two systemic diseases, and three (2.0%) patients had three systemic diseases. Concomitant systemic diseases were hypertension in 33 (22.0%) patients, diabetes mellitus in 24 (16.0%) patients, hyperlipidemia in eight (5.3%) patients, hypothyroidism in four (2.7%) patients, chronic obstructive pulmonary disease (COPD) in two (1.3%) patients, Parkinson’s disease in two (1.3%) patients, epilepsy in one (0.7%) patient, and xeroderma pigmentosum in one (0.7%) patient (Table 1).

**Table 1:** Distribution of comorbid systemic diseases in the three groups (non-melanoma skin cancer, psoriasis and vitiligo) 41 (27.3%) patients had one systemic disease, 13 (8.7%) patients had two systemic diseases, and three (2.0%) patients had three systemic diseases.

Comorbid systemic disease	Non-melanoma skin cancer, n (%)	Psoriasis, n (%)	Vitiligo, n (%)	Total, n (%)
Hypertension	17 (34.0)	8 (16.0)	8 (16.0)	33 (22.0)
Diabetes mellitus	10 (20.0)	10 (20.0)	4 (8.0)	24 (16.0)
Hyperlipidemia	1 (2.0)	5 (10.0)	2 (4.0)	8 (5.3)
Hypothyroidism	0 (0)	1 (2.0)	3 (6.0)	4 (2.7)
Chronic obstructive pulmonary disease ​​​​​​(COPD)	0 (0)	2 (4.0)	0 (0)	2 (1.3)
Parkinson’s disease	0 (0)	1 (2.0)	1 (2.0)	2 (1.3)
Epilepsy	0 (0)	1 (2.0)	0 (0)	1 (0.7)
Xeroderma pigmentosum	1 (2.0)	0 (0)	0 (0)	1 (0.7)

In those patients, all comorbid diseases were well-controlled and when the patients were grouped according to the presence or absence of concomitant systemic disease, no statistical difference was found between the groups in terms of concomitant systemic disease (Chi-square test; P=0.361) (Table 2).

**Table 2:** Distribution of patient groups according to the presence of comorbid systemic diseases Chi-square=2.04; SD=2; P=0.361.

Comorbid systemic disease	Non-melanoma skin cancer, n (%)	Psoriasis, n (%)	Vitiligo, n (%)	Total, n (%)
Present	21 (36.8)	21 (36.8)	15 (26.3)	57 (100)
Absent	29 (31.2)	29 (31.2)	35 (37.6)	93 (100)
Total	50 (33.3)	50 (33.3)	50 (33.3)	150 (100)

The clinical features and findings of patients with non-melanoma skin cancers, psoriasis, and vitiligo are presented in Table 3.

**Table 3:** The clinical features and findings of patients with non-melanoma skin cancers, psoriasis, and vitiligo

	Non-melanoma skin cancer (n=50)
Duration of disease (months)	22.2±27.6
Non-melanoma skin cancer type	
Basal cell carcinoma (BCC)	33 (66%)
Squamous cell carcinoma (SCC)	17 (34%)
Basal cell carcinoma (BCC) type	
Nodular	25 (75.7%)
Superficial	6 (18.2%)
Pigmented	2 (6.1%)
Location	
Nose	
Yes	20 (40%)
No	30 (60%)
Eyelid	
Yes	3 (6%)
No	47 (94%)
Cheek	
Yes	8 (16%)
No	42 (84%)
Temporal	
Yes	2 (4%)
No	48 (96%)
Scalp	
Yes	3 (6%)
No	47 (94%)
Lips	
Yes	8 (16%)
No	42 (84%)
Ear	
Yes	3 (6%)
No	47 (94%)
Neck	
Yes	1 (2%)
No	49 (98%)
Other	
Yes	2 (4%)
No	48 (96%)
	Psoriasis (n=50)
Duration of disease (months)	129.5±122.9
Psoriasis Area and Severity Index (PASI) score	9.6±7.3
Psoriasis type	
Guttate	7 (14%)
Vulgaris	39 (78%)
Pustular	4 (8%)
Location	
Scalp	
Yes	27 (54%)
No	23 (46%)
Face	
Yes	7 (14%)
No	43 (86%)
Neck	
Yes	7 (14%)
No	43 (86%)
Nail	
Yes	14 (28%)
No	36 (72%)
Body	
Yes	32 (64%)
No	28 (36%)
Upper extremity	
Yes	46 (92%)
No	4 (8%)
Lower extremity	
Yes	48 (96%)
No	2 (4%)
Joint involvement	
Yes	11 (22%)
No	39 (78%)
	Vitiligo (n=50)
Duration of disease (months)	121±114.6
Vitiligo type	
Localized	7 (14%)
Generalized	42 (84%)
Segmental	1 (2%)
Location	
Face	
Yes	35 (70%)
No	15 (30%)
Body	
Yes	25 (50%)
No	25 (50%)
Genital	
Yes	17 (34%)
No	33 (66%)
Upper extremity	
Yes	37 (74%)
No	13 (36%)
Lower extremity	
Yes	36 (72%)
No	14 (28%)

There was no statistically significant difference between BCC and SCC patients evaluated with the EORTC QLQ-C30 general cancer scale, WHOQOL-BREF TR, and with DLQI in terms of QoL in any dimension; the same was true for BCC subgroups (Mann-Whitney U test; P>0.05) (Table 4).

**Table 4:** Comparison of quality of life between BCC and SCC patients and in BCC subgroups with EORTC QLQ-C30 Values were presented as “mean±SD”; only the p values for the comparison between groups were presented. *Mann-Whitney U.

European Organization for Research and Treatment of Cancer QLQ-Core 30 (EORTC QLQ-C30)	Non-melanoma skin cancer (n=50 patients)	Difference between basal cell carcinoma (BCC) and squamous cell carcinoma (SCC), P*	Difference between BCC subgroups, P*
Global health status quality of life (QoL)	59.8±25.8	P=0.757	P=0.470
Functional subscales			
Physical functioning	70.9±23.3	P=0.570	P=0.918
Role functioning	83.3±24.1	P=0.684	P=0.606
Emotional functioning	65.0±29.5	P=0.187	P=0.374
Cognitive functioning	75.3±20.6	P=0.833	P=0.420
Social functioning	76.0±27.0	P=0.446	P=0.984
Symptom subscales			
Fatigue	30.9±22.6	P=0.566	P=0.397
Nausea and vomiting	7.7±18.2	P=0.265	P=0.470
Pain	32.0±27.3	P=0.675	P=0.522
Dyspnea	21.3±28.4	P=0.221	P=0.853
Sleep disturbance	27.3±30.6	P=0.162	P=0.606
Appetite loss	19.3±28.6	P=0.795	P=0.352
Constipation	13.3±27.8	P=0.150	P=0.290
Diarrhea	2.7±9.1	P=0.486	P=0.726
Financial impact	25.3±34.1	P=1.000	P=0.150

QoL was statistically different in NMSC, psoriasis, and vitiligo groups (ANOVA; P<0.05). (Tables 5, 6). When the QoL measured by the DLQI was compared in the non-melanoma skin cancer, psoriasis, and vitiligo groups, the QoL in non-melanoma skin cancer and psoriasis patients was similar in the total DLQI, symptoms and feelings, and work and school subscales, and deteriorated more than in vitiligo patients (post-hoc Tukey B). In the dimensions of daily activities and personal relationships, QoL deteriorated more in psoriasis patients than in vitiligo patients (post-hoc Tukey B). In the dimensions of leisure and treatment, quality of life deteriorated the most in psoriasis patients, followed by non-melanoma skin cancer and vitiligo patients (post-hoc Tukey B) (Table 5).

**Table 5:** Comparison of quality of life measured by Dermatology Life Quality Index (DLQI) in patient groups (values were presented as mean±sd) *ANOVA. **According to post-hoc analysis with Tukey B. a: DLQI index was similar between groups 1 and 2; and both groups had significantly higher DLQI index than group 3. b: Group 2 had significantly higher DLQI index compared to group 3; but no differences between groups 1 and 3; and also between groups 1 and 2. c: Group 2 had significantly higher DLQI index compared to group 1; and both had significantly higher DLQI index than group 3. d: DLQI index was similar between groups 1 and 2; and both were significantly higher DLQI index than group 3. e: Group 2 had significantly higher DLQI index compared to group 3; but no differences between groups 1 and 3; and also between groups 1 and 2. f: Group 2 had significantly higher DLQI index compared to group 1; and both had significantly higher DLQI index than group 3. g: DLQI index was similar between groups 1 and 2; and both were significantly higher DLQI index than group 3.

	Dermatology Life Quality Index (DLQI)			P* (post hoc, Tukey B)**
Domain	Non-Melanoma Skin Cancer (n=50) (group 1)	Psoriasis (n=50) (group 2)	Vitiligo (n=50) (group 3)	
Symptoms and feelings	2.3±1.5	2.9±1.7	1.6±1.0	P=0.000* a**
Daily activities	1.4±1.5	1.9±1.8	1.1±1.2	P=0.036* b**
Leisure	1.3±1.5	1.9±1.9	0.6±0.8	P=0.000* c*
Work and school	2.1±1.4	2.0±1.4	1.1±1.5	P=0.001* d**
Personal relationships	1.4±1.6	1.7±1.8	0.9±1.1	P=0.047* e**
Treatment	0.7±0.8	1.1±0.9	0.3±0.5	P=0.000* f**
Total DLQI	9.2±6.7	11.6±8.0	5.7±3.8	P=0.000* g**

When the QoL measured by WHOQOL-BREF TR was compared in patients with non-melanoma skin cancer, psoriasis, and vitiligo, the QoL in NMSC and psoriasis patients was similar in the physical health and social relationships domain dimensions and deteriorated more than in vitiligo patients (post-hoc Tukey B). In the Environment Domain-Tr (+National Social Pressure) dimension, QoL deteriorated more in non-melanoma skin cancer patients than in vitiligo patients (post-hoc Tukey B). In the psychological health domain dimension, there was no statistically significant difference between the groups in terms of QoL (post-hoc Tukey B) (Table 6).

**Table 6:** Comparison of quality of life measured by World Health Organization Quality of Life-BREF, Turkish version (WHOQOL-BREF TR) (0-20) in patient groups (values were presented as mean±SD) *ANOVA. ** Post-Hoc Tukey B. a: WHOQOL-BREF TR index was similar between groups 1 and 2; and both were significantly higher WHOQOL-BREF TR index than group 3. b: WHOQOL-BREF TR index was similar between groups 1 and 2; and both had significantly higher WHOQOL-BREF TR index than group 3. c: Group 1 had significantly higher WHOQOL-BREF TR index compared to group 3; but no differences between groups 2 and 3; and also between groups 1 and 2.

Domain	World Health Organization Quality of Life Short Form (BREF), Turkish version (WHOQOL-BREF TR) Score			P* (post hoc, Tukey B)**
	Non-melanoma skin cancer (n=50) (Group 1)	Psoriasis (n=50) (Group 2)	Vitiligo (n=50) (Group 3)	
Physical health	13.4±3.2	13.9±2.7	16.2±2.1	P=0.000* a**
Psychological health	12.9±3.1	12.8±3.1	13.5±2.0	P=0.359*
Social relationships	13.2±3.2	13.9±3.6	15.3±1.9	P=0.002* b**
Environment-Tr (+National Social Pressure)	13.0±2.8	13.3±2.5	14.4±1.9	P=0.015* c **

In addition, when the DLQI scores of patients with a PASI score of 10 and above and those with a PASI score of less than 10 were compared, patients with a PASI score of 10 and above had more impaired QoL in total DLQI and leisure and personal relationships sub-dimensions (Mann-Whitney U test; P=0.043, P=0.028, P=0.046). (Table 7).

**Table 7:** Comparison of Dermatology Life Quality Index (DLQI) scores in psoriasis patients with Psoriasis Area and Severity Index (PASI) scores of 10 and above (Group 1) and those with PASI scores below 10 (Group 2) * Mann-Whitney U. Highlighted p-values indicate P<0.05. The values were presented as “mean±standard deviation”. Four patients with pustular psoriasis were excluded from analysis (PASI score was not calculated for those patients).

Domain	Dermatology Life Quality Index (DLQI) Scores		
	Group 1 (n=21)	Group 2 (n=25)	P*
Symptoms and feelings	3.1±1.7	2.5±1.6	0.177
Daily activities	2.2±1.9	1.6±1.7	0.266
Leisure	2.5±1.7	1.4±1.8	0.028
Work and school	2.3±1.3	1.8±1.5	0.248
Personal relationships	2.3±2.0	1.2±1.6	0.046
Treatment	1.2±0.8	0.9±0.9	0.132
Total Dermatology Life Quality Index (DLQI)	13.7±7.9	9.4±7.5	0.043

When the relationship between disease duration and QoL in patient groups was examined, a negative correlation was found between disease duration and DLQI work and school scores in non-melanoma skin cancer patients. In other words, as the disease duration increases, DLQI work and school scores decrease, and therefore, quality of life increases in this dimension (Pearson correlation test; r=-0.25, P=0.002).

In vitiligo patients, a negative correlation was found between disease duration and DLQI work and school scores. In other words, as the disease duration increases, DLQI work and school scores decrease, QoL in this dimension increases (Pearson correlation test; r=-0.39, P=0.006). In addition, a negative correlation was found between disease duration and WHOQOL-BREF TR physical health domain scores. In other words, as the duration of the disease increases, WHOQOL-BREF TR physical health domain scores decrease, and therefore, QoL in this dimension decreases (Pearson correlation test; r=-0.44, P=0.001).

No correlation was found between disease duration and QoL in psoriasis patients.

A sub-analysis was conducted to compare the QoL scores based on lesion location (visible vs. non-visible areas) in the non-melanoma skin cancer, psoriasis, and vitiligo groups. This analysis revealed that only the treatment dimension of the DLQI was significantly more impaired in patients with lesions in non-visible areas (Student’s t-test; P=0.001), while no significant differences were observed in other DLQI dimensions or in any domains of the WHOQOL-BREF TR.

## Discussion

In cancer research, QoL has been considered as an important outcome. Particularly, it has been demonstrated that cancer is linked to a wide range of psychological side effects, including sensitivity, anxiety, and despair [8]. Psoriasis is well-known as a disease that severely impacts QoL [5]. Our study revealed that NMSC had a similar impact on QoL as psoriasis. Although BCC and SCC have modest mortality rates, their common occurrence on the head and neck can result in severe morbidity due to disfigurement. Both the presence and treatment of NMSC can cause visible scarring and contribute to both mental and physical impairment. As a result, QoL should be considered an important outcome in the management of NMSC.

Studies have shown that most general QoL scales and dermatology-specific QoL scales are not suitable for indicating QoL in NMSCs [9,10]. There are QoL questionnaires specifically designed for NMSC patients, such as the Skin Cancer Index (SCI) and the Skin Cancer Quality of Life Impact Tool (SCQOLIT) [11,12]. Nevertheless, these QoL questionnaires were not validated yet in Turkey, as this study was conducted [13]. In our study, QoL in NMSC was evaluated with DLQI, a dermatology-specific scale, WHOQOL-BREF TR, a general health-specific QoL scale, and EORTC QLQ-C30, a general cancer scale before treatment. In our study, in 50 NMSC patients (33 (66%) BCC and 17 (34%) SCC), most of the lesions were located in the nose (40%); the mean total DLQI score before treatment was 9.2±6.7. Rhee et al. evaluated the QoL in NMSC with DLQI before and four months after treatment and showed that the majority of lesions were located in the nose (37%) in a total of 121 patients, 103 (85%) BCC and 13 (16%) SCC, and the mean total DLQI score before treatment was 2.4±2.7 (n=121) and after treatment was 1.7±2.9 (n=101) [8]. In both studies, the mean age of the patients and the location of the majority of the lesions are similar, but the mean total DLQI score before treatment is greater in our study. This could be related to the fact that our study included more SCC patients and hence their QoL may have deteriorated more. In our study, no statistically significant difference was found between BCC and SCC patients evaluated with the EORTC QLQ-C30 general cancer scale, DLQI, and WHOQOL-BREF TR in terms of QoL in any dimension, and the same was true for BCC subgroups (Mann-Whitney U test; P>0.05).

In a study conducted by Steinbauer et al., the total DLQI scores were divided into five categories in a group of 52 patients, including BCC (69%, n=36), SCC (15%, n=8), actinic keratosis (25%, n=13), and Bowen’s disease (8%, n=4) in NMSCs: from no impairment to very much impairment. Moderate or severe impairment was found in 31% of the patients, whereas no or mild impairment was found in the remaining 70%. When demographic and clinical variables were compared with QoL, no correlation was found between age, gender, location of lesions, or type of disease and QoL [14]. Thus, there was no statistically significant difference in QoL between NMSCs in this study or in our study. Steinbauer et al. concluded that in future studies, in addition to the DLQI, a dermatology-specific scale, and a general health-specific QoL scale such as the 36-item Short Form Survey (SF-36) or the EORTC QLQ-C30 cancer scale should be used [14].

In our study, in addition to DLQI, the EORTC QLQ-C30 general cancer scale, and WHOQOL-BREF TR were used to assess QoL, but no statistically significant difference was found between BCC and SCC patients or between BCC clinical types in any dimension.

Radtke et al. assessed the quality of life of 1023 vitiligo patients and 1511 psoriasis patients using the DLQI (a dermatology-specific scale) and Euro-QoI 5D (EQ-5D) (a general health scale). Unlike our study, they discovered that as disease duration increased, quality of life decreased. Similar to our findings, vitiligo patients had a lower mean total DLQI score (7.0±5.9) than those with psoriasis (8.6±7.1) [15].

Similarly, in the study of Ongenae et al., the mean total DLQI score was found to be lower in the vitiligo group (4.95) compared to the psoriasis group (6.26) [16]. In our study, QoL was compared in NMSC (n=50), psoriasis (n=50), and vitiligo (n=50) groups using DLQI, a dermatology-specific scale, and WHOQOL-BREF TR, a general health scale. We found that QoL was statistically different in the NMSC, psoriasis, and vitiligo groups (ANOVA; P<0.05). The DLQI-measured QoL in NMSC and psoriasis patients was similar in total DLQI, symptoms and feelings, work and school sub-dimensions, and deteriorated more than in vitiligo patients. Additionally, in the dimensions of daily activities and personal relationships, QoL was found to be more impaired in psoriasis patients than in vitiligo patients. In the dimensions of leisure and treatment, QoL deteriorated the most in psoriasis patients, followed by NMSC and vitiligo patients, respectively. QoL assessed with WHOQOL-BREF TR was similar in the physical health domain and social relationships domain dimensions in NMSC and psoriasis patients, and deteriorated more than in vitiligo patients. QoL in NMSC patients decreased more than in vitiligo patients in the environment domain-Tr (+National Social Pressure) dimension. In terms of QoL, there was no statistically significant difference between the groups in the psychological health domain dimension. In our study, similar results were obtained in all patient groups for QoL measured by DLQI and WHOQOL-BREF TR, indicating that DLQI, a dermatology-specific scale, can be used in conjunction with WHOQOL-BREF TR, a general health-specific scale, in comparing the QoL of diseases in dermatology.

In their study investigating the effects of psoriasis on QoL, Weiss et al. found no correlation between PASI scores and Satisfaction with Life Scale (SWLS), EQ-5D, and SF-36 scales. With the results of the study, the researchers concluded that in future studies, a QoL scale that also measures the psychosocial dimension of the disease should be used in addition to scales such as the PASI, which measures only the physical dimension of psoriasis severity [17]. Shikiar et al., according to the results of a phase II study investigating the sensitivity of DLQI, SF-36, and EQ-5D QoL scales in psoriasis patients, showed that DLQI was the most sensitive scale. Changes in DLQI scores were correlated with changes in PASI scores throughout the study. In addition, this study showed that among the QoL scales, DLQI is the only QoL scale that is equally sensitive to PASI scores in psoriasis patients [18].

Aghaei et al., in their study investigating the effect of the disease on QoL in 125 psoriasis patients in Iran using the Psoriasis Disability Index (PDI) and DLQI scales, found a statistically significant strong correlation between mean PDI and DLQI scores and PASI scores; and as PASI scores increased, PDI and DLQI scores increased, indicating deterioration in QoL [19]. Similarly, in our study, the mean DLQI score was 11.6±8.0 and the PASI score was 9.6±7.3. In addition, when the DLQI scores of patients with a PASI score of 10 and above and those with a PASI score of less than 10 were compared, patients with a PASI score of 10 and above had more impaired QoL in total DLQI and leisure and personal relationships sub-dimensions (Mann-Whitney U test; P=0.043, P=0.028, P=0.046). In other words, a correlation was observed between PASI scores and DLQI scores. Our findings confirm previously reported correlations between PASI and DLQI scores in psoriasis patients.

In vitiligo patients, a negative correlation was found between disease duration and DLQI work and school scores. In other words, as the disease duration increases, DLQI work and school scores decrease, and therefore, QoL in this dimension increases (Pearson correlation test; r=-0.39, P=0.006). This observed improvement may reflect patients’ adaptation to their condition over time, whereby acceptance of the disease and lifestyle modifications help mitigate its perceived impact on work and school activities. In addition, a negative correlation was found between disease duration and WHOQOL-BREF TR physical health domain scores. In other words, as the duration of the disease increases, WHOQOL-BREF TR physical health domain scores decrease, and therefore, QoL in this dimension decreases (Pearson correlation test; r=-0.44, P=0.001).

Our findings align with previous research comparing the quality of life across different chronic dermatologic conditions. Ghajarzadeh et al. reported significantly higher DLQI scores and lower SF-36 scores in psoriasis patients compared to those with vitiligo or alopecia areata, indicating poorer QoL in psoriasis. They also demonstrated a moderate correlation between DLQI and depressive symptoms in all disease groups, underscoring the link between psychological burden and QoL [20]. Similarly, Siddiqui et al. highlighted the profound psychosocial impact of pediatric psoriasis and vitiligo, noting their strong association with mental health challenges [21]. Their review further indicated that greater disease severity worsens QoL, while longer disease duration may improve physical functioning, a finding that resonates with our observation of improved DLQI work and school scores over time in vitiligo patients. Together, these studies support the importance of integrating psychosocial assessment and targeted interventions, such as psychological counseling or peer support programs, into the management of chronic skin diseases.

Given the comparable life disruption observed in NMSC and psoriasis, targeted self-care strategies may help reduce QoL impairment in both groups. These could include regular skin self-examination, strict sun protection measures, and adherence to prescribed topical or systemic treatments. Furthermore, incorporating psychosocial screening into routine dermatology visits and developing tailored support programs may enable early identification of patients at higher risk for significant life disruption, thereby facilitating timely and effective interventions.

The main limitations of our study were the short duration of the study, which lasted only six months, and the resulting small number of patients in each group. The observational nature of the study does not allow for causal inference. No matching of age or disease duration was performed across groups, which may act as potential confounders. In our study, QoL was assessed before treatment in NMSC patients, but it was not measured after treatment. Another limitation was that we could not use NMSC patient-specific QoL scales like SCI and SCQOLIT as they were not validated in Turkey.

## Conclusions

Our study revealed that patients with NMSC reported similar QoL scores to those with psoriasis, which is well-known as a disease that severely impacts quality of life. As a result, QoL should be a key consideration in managing NMSC, and future treatments should focus on approaches that preserve or enhance it.

While numerous studies have evaluated QoL in NMSC, this study is the first to employ a combination of dermatology-specific and general health QoL scales to compare NMSC, psoriasis, and vitiligo. In our study, the EORTC QLQ-C30 general cancer scale, the DLQI, and the WHOQOL-BREF TR were used to assess QoL, but no statistically significant difference was found between BCC and SCC patients or between BCC clinical types in any dimension. Since the existing scales are not sensitive to this population, the results of our study and previous studies point to the need for a disease-specific QoL scale.

In our study, we found that QoL was statistically different among the NMSC, psoriasis, and vitiligo groups. Obtaining similar QoL scores in all groups by DLQI and the WHOQOL-BREF-TR questionnaire may indicate that the WHOQOL-BREF TR scale (a scale to assess QoL of general health) can be used in conjunction with the DLQI (a specific questionnaire to assess QoL in dermatologic diseases) scale to assess QoL in dermatologic diseases.
